# Embedded NMR Sensor to Monitor Compressive Strength Development and Pore Size Distribution in Hydrating Concrete

**DOI:** 10.3390/s131215985

**Published:** 2013-11-25

**Authors:** Floriberto Díaz-Díaz, Prisciliano F. de J. Cano-Barrita, Bruce J. Balcom, Sergio E. Solís-Nájera, Alfredo O. Rodríguez

**Affiliations:** 1 Instituto Politécnico Nacional-CIIDIR Unidad Oaxaca, Hornos No. 1003, Sta. Cruz Xoxocotlán, Oaxaca 71230, Mexico; E-Mail: floribertodiazdiaz@gmail.com; 2 MRI Centre, Department of Physics, University of New Brunswick, Fredericton, NB E3B 5A3, Canada; 3 Facultad de Ciencias, Universidad Nacional Autónoma de México, Mexico Distrito Federal 04510, Mexico; E-Mail: solisnajera@ciencias.unam.mx; 4 Departmento de Ingeniería Eléctrica, Universidad Autónoma Metropolitana Iztapalapa, Mexico Distrito Federal 09340, Mexico; E-Mail: arog@xanum.uam.mx

**Keywords:** embedded NMR sensor, RF coil, external tuning circuit, T_2_ relaxation time, cement paste, concrete, compressive strength, pore size distribution

## Abstract

In cement-based materials porosity plays an important role in determining their mechanical and transport properties. This paper describes an improved low–cost embeddable miniature NMR sensor capable of non-destructively measuring evaporable water loss and porosity refinement in low and high water-to-cement ratio cement-based materials. The sensor consists of two NdFeB magnets having their North and South poles facing each other, separated by 7 mm to allow space for a Faraday cage containing a Teflon tube and an ellipsoidal RF coil. To account for magnetic field changes due to temperature variations, and/or the presence of steel rebars, or frequency variation due to sample impedance, an external tuning circuit was employed. The sensor performance was evaluated by analyzing the transverse magnetization decay obtained with a CPMG measurement from different materials, such as a polymer phantom, fresh white and grey cement pastes with different w/c ratios and concrete with low (0.30) and high (0.6) w/c ratios. The results indicated that the sensor is capable of detecting changes in water content in fresh cement pastes and porosity refinement caused by cement hydration in hardened materials, even if they are prepared with a low w/c ratio (w/c = 0.30). The short lifetime component of the transverse relaxation rate is directly proportional to the compressive strength of concrete determined by destructive testing. The r^2^ (0.97) from the linear relationship observed is similar to that obtained using T_2_ data from a commercial Oxford Instruments 12.9 MHz spectrometer.

## 1. Introduction

In Portland cement concrete, porosity plays an important role in determining its mechanical and durability properties. Porosity depends on the water-to-cement ratio and the degree of hydration of the cement paste [1]. On exposure to the environment the material undergoes changes that lead to micro-cracking, increasing the permeability of concrete to water, which is a medium for transport of other aggressive species such as chloride ions and CO_2_ [1]. Therefore, monitoring the moisture condition and microstructural changes in concrete are important from durability and mechanical strength points of view.

The moisture condition is normally evaluated by using relative humidity sensors that must be installed by drilling into the structural member [2]. Information obtained in this way is of limited use, unless the sorption/desorption isotherm is known for the material to determine the moisture content corresponding to a specific relative humidity. On the other hand, compressive strength of concrete is normally determined by destructive testing of cylinders cast for this purpose or by coring the structure to determine the “*in situ*” compressive strength. Traditionally, the rebound hammer and the ultrasonic pulse velocity are used to estimate the compressive strength of concrete in the field provided a calibration curve is first determined. There are however several additional factors affecting the measurements such as moisture content, type and amount of aggregates, and the presence and direction of the reinforcing steel [3].

Nuclear magnetic resonance (NMR) is a non-destructive and non-invasive technique that has been used in the laboratory to study cement-based materials [4–6]. To extend the possibilities of NMR to study these materials in the field, portable systems such as the NMR MOUSE [7,8] and the NMR MOLE [9] have been developed but the measurements are restricted to a depth of a few millimeters below the surface. Embedded miniature NMR sensors have been used to monitor changes in evaporable water and pore refinement in high water/cement ratio Portland cement mortar [10]. However, use of these sensors in materials having a low water/cement ratio (low porosity) did not provide useful NMR signal because of low signal intensity.

This paper presents an improved embedded NMR sensor capable of measuring microstructural and evaporable water changes occurring in concrete materials having high and low water/cement ratios, such as those used in the construction industry for ordinary and special applications, respectively.

## 2. Experimental Section

### 2.1. Sensor Design and Construction

**Figure 1.**
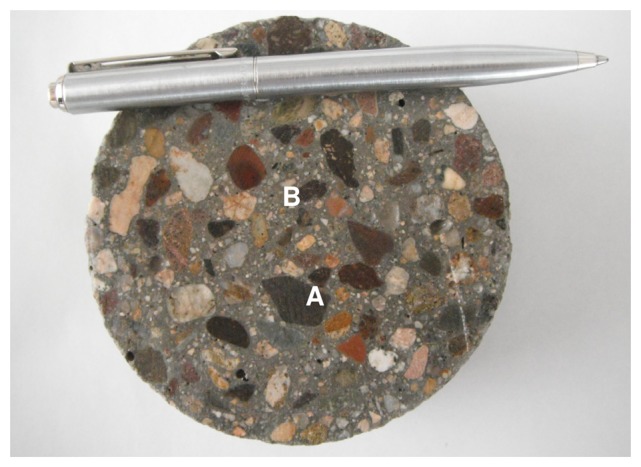
Piece of hydraulic concrete showing coarse aggregates (**A**) and mortar (**B**). Mortar is composed of cement paste and fine aggregates (particle size < 4.75 mm).

Concrete is essentially a material containing hydrated Portland cement paste and aggregates (Figure 1). Since hydration of the cement paste is the process leading to changes in mechanical and transport properties of concrete, it is the part of the material that the sensor must be capable of monitoring. The sensor design considered the fact that aggregates, especially normal density coarse aggregates (>4.75 mm), may drastically reduce the amplitude of the NMR signal if they are located within the sensitive region of the sensor. These aggregates have low water absorption (less than 2%). Even if considering lightweight aggregates with much higher water absorption, the NMR signal detected would not be of interest since it does not reflect changes occurring in the cement paste caused by hydration.

**Figure 2.**
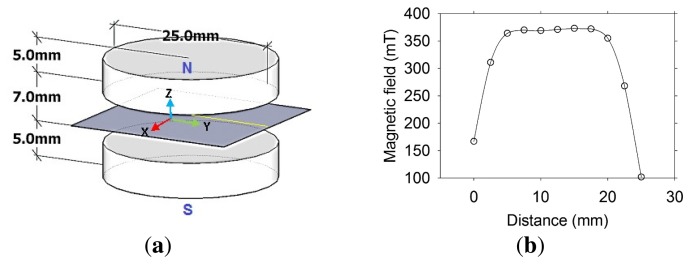
(**a**) Magnet arrangement for the sensor; (**b**) Measured Z magnetic field component along the Y-axis.

Therefore, the sensor has to prevent aggregate particles from entering into its sensitive region. Several magnet arrangements were explored and the Z magnetic field component along the Y-axis (Figure 2) was measured to select those providing the highest and the most homogeneous magnetic fields [11]. The best arrangement was chosen based on the highest signal to noise ratio (SNR) obtained by measuring the transverse magnetization decay of a polymer phantom using the CPMG technique [12].

The magnet arrangement selected consists of two circular grade 35 NdFeB magnets, measuring 25 mm in diameter and 5 mm in thickness, with opposite poles facing (Figure 2a). This had an additional advantage of increasing the magnetic field strength and homogeneity that in turn increases sensitivity of the sensor. Figure 2b shows the magnetic field measured at the middle of the distance between the magnets. The measurement was performed by manually displacing a Gaussmeter at 2.5 mm intervals along the Y-axis. For this particular pair of magnets, the magnetic field was 0.38 T and the Larmor frequency for ^1^H was 16.18 MHz.

**Figure 3.**
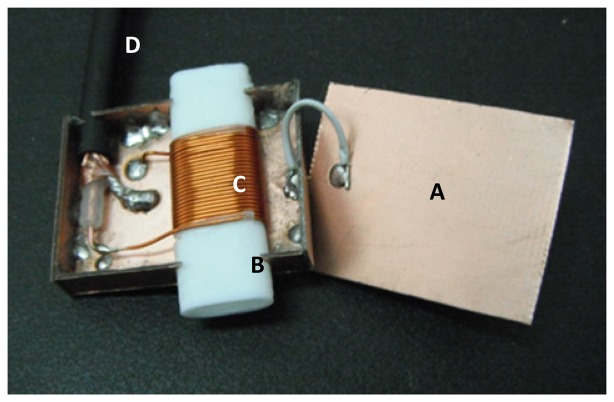
Faraday cage (**A**) containing Teflon tube (**B**), ellipsoidal RF coil (**C**), and RG58 coaxial cable (**D**).

The separation distance between the magnets was 7 mm to allow space for a Faraday cage (Figure 3) made of a phenolic printed circuit board plate, while preventing coarse aggregates from entering into the sensitive region of the sensor. The Faraday cage was used to reduce the influence of external noise on the NMR signal. During preliminary evaluation of the design, it was observed that when the sensor was introduced in the cement paste, there were changes in the tuning frequency and in the impedance of the RF coil. This was due to the influence of external impedance (fresh cement paste) over the total impedance of the RF coil, which hindered external tuning. To reduce this problem, a Teflon tube with ellipsoidal cross section measuring 11 mm in major axis, 5 mm in minor axis and 30 mm in length was used. The tube served also as a mold for the RF coil, which had 22 turns of 20 AWG copper wire. The length of the coil was 23 mm and an inductance of 2.8 μH. The length of the RG58 coaxial cable was 1.17 m (λ/16) with an effective capacitance of 110.8 pF.

**Figure 4.**
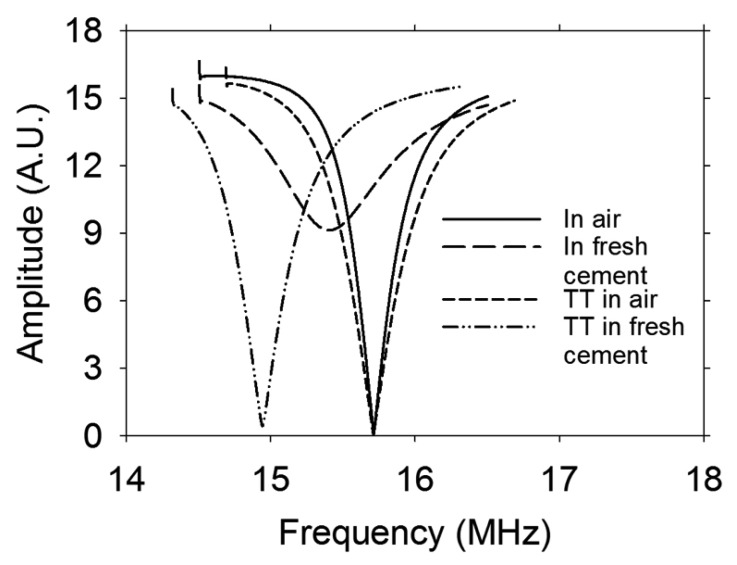
Frequency response of the sensor outside and inside the fresh cement paste w/c ratio = 0.60. TT means sensor with Teflon tube.

Figure 4 shows the response in frequency when the sensor without the Teflon tube is immersed in fresh cement paste. There is a change in both frequency and impedance. However, if the Teflon tube is used (could also be a glass tube) there is only a change in frequency. The effect of coupling in impedance and the change in frequency of the coil when it is embedded in the cement paste depends on the characteristics of the material, such as polarity and the dielectric constant. It is observed that the Teflon tube eliminates the impedance displacement when the sensor is embedded in the cement paste, although it does not avoid changes in frequency. This change in frequency was accommodated using the external tuning circuit described next.

**Figure 5.**
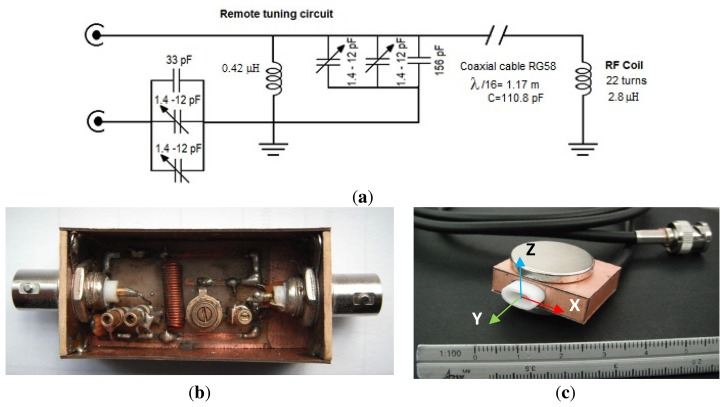
(**a**) Circuit diagram of the sensor; (**b**) External tuning circuit (55 mm in length, 25 mm in width, and 20 mm in height); (**c**) NMR sensor constructed.

As shown in Figure 5a, the coil design does not include capacitors within the sensor; rather the tuning (16.18 MHz) was performed through a remote tuning circuit (Figure 5b). The purpose of this remote tuning circuit was to re-tune the RF coil after the sensor is embedded to accommodate frequency changes due to sample impedance, changes in temperature and/or the presence of steel rebars that influence the static magnetic field, which in turn changes the Larmor frequency. The main advantage of the external tuning circuit is the possibility of retuning the RF coil once the sensor is embedded, compared to conventional tuning-matching circuits. The function of the additional inductance (0.42 μH) in the external tuning circuit is to adjust the resonance frequency of the RF coil. It is connected in parallel to the total inductance (coaxial cable and RF coil), therefore the equivalent inductance obtained is lower and the frequency is increased [13]. The entire sensor was covered with a layer of water resistant epoxy resin (Figure 5c).

### 2.2. Sensor Characterization

#### 2.2.1. NMR Measuring Technique

The CPMG technique [12] was used to obtain the transverse magnetization decay of protons from a polymer phantom and from evaporable water in fresh and hardened cement paste and concrete.

Table 1 provides the CPMG parameters used to determine the transverse magnetization decay of a polymer phantom, which possesses a bi-exponential decay with a long lifetime component of 20 ms and a short lifetime component of 4.5 ms. The SNR was obtained as the ratio of the average amplitude of the first three points of the CPMG envelope divided by the standard deviation of the last ten points where the signal has completely decayed. The coefficient of variation of the CPMG amplitude and the T_2_ decay constant obtained by performing seven measurements with the same sensor and the same polymer phantom under temperature controlled conditions was less than 1%.

Table 2 provides the CPMG parameters used for the measurements in cement pastes and concretes. These parameters were used according to the type, w/c ratio and age of the samples, so that the changes in microstructure produced by cement hydration and reflected in the relaxation times could be followed. For instance, the minimum number of scans, the maximum echo time, the maximum number of echoes, and the maximum repetition time, were used at early ages, when the T_2_ relaxation times were on the order of few milliseconds.

#### 2.2.2. NMR Sensors to Characterize Fresh and Hardened Portland Cement Pastes

Ordinary and white Portland cement were used to prepare the cement pastes. White cement was used because of its lower iron content compared to grey cement (Table 3). Tap water was used to prepare the cement pastes.

Seven cement pastes were prepared at w/c ratios by weight of 0.30, 0.35, 0.40, 0.45, 0.50, 0.55 and 0.60. Immediately the sensor was immersed in the fresh cement paste and the CPMG technique was used to obtain the transverse magnetization decay, which was in all cases best fit to a mono-exponential decay function to determine the NMR signal amplitude and the T_2_ decay constant.

Samples from the fresh cement pastes were only taken from those having w/c ratios of 0.30, 0.40, 0.50 and 0.60. They were introduced in small plastic tubes with appropriate size to fit into the sensor and sealed to avoid ingress or loss of moisture. The transverse magnetization decay was determined in the hardened state at 1, 3, 7, 14, and 28 days of age. It was verified that the plastic tubes did not provide any NMR signal.

#### 2.2.3. NMR Sensors Embedded in Hydraulic Concrete Cylinders

Four sensors were built to monitor changes in the transverse magnetization decay during hydration of cement in concrete. Two mixes with w/c ratios of 0.30 and 0.60 were prepared with the proportions given in Table 4. The materials used were river sand having a fineness modulus of 2.9, specific gravity of 2.65, and absorption of 1.73%. The gravel had a maximum size of ¾ inch, specific gravity of 2.68, and absorption of 1.51%. The chemical composition of the fine aggregate used is given in Table 1. Tap water was used to prepare the concrete mixtures.

**Figure 6.**
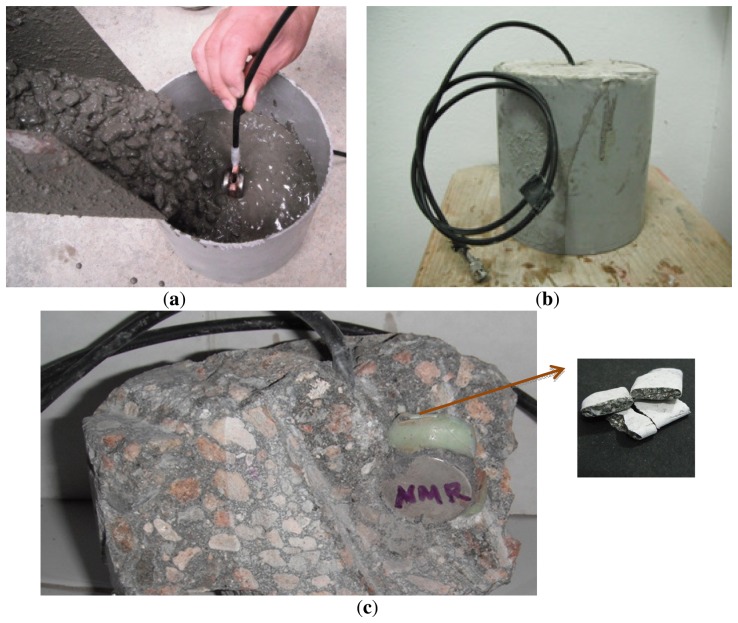
(**a**) Sensor embedded in fresh concrete during external vibration of the material, which made it easy to introduce cement paste and small fine aggregate particles into the sensor; (**b**) Sensor in a hardened concrete cylinder; (**c**) After breaking a hardened specimen to extract the sensor, it was observed that only cement paste and some fine aggregate particles (size < 1 mm) had penetrated into the sensor and filled the measurement volume.

From each concrete mixture two cylinders measuring 150 mm in diameter and 150 mm in height were cast and immediately one sensor was embedded to a half of the total height of the cylinder (Figure 6a). Before embedding the sensors in the concrete specimens, the SNR for each sensor was determined by obtaining the NMR signal from a polymer phantom. Seven specimens in triplicate measuring 100 mm in diameter and 200 mm in height were cast for compressive strength testing at 1, 3, 7, 14, 21, 28 and 56 days of age (a total of 21 cylinders). After 24 h, all the specimens were removed from the molds and stored in a moist room at 22 ± 3 °C until testing. At this time, the cylinders with the embedded sensors (Figure 6b) were non-destructively tested using a portable Magritek Kea^2^ spectrometer to obtain the transverse magnetization decay using the CPMG technique. After each cylinder was destructively tested under compression, a sample from the center was taken for NMR measurements in an Oxford Instruments Maran DRX-HF 12/50 spectrometer (Oxford Instruments, Abingdon, UK) at 12.9 MHz, to compare the results obtained with the embedded sensors.

#### 2.2.4. Influence of Temperature and Reinforcing Steel Bars on Sensor Resonant Frequency

To measure the effects of temperature on sensor resonant frequency, it was located in a controlled environment at 17.5, 20 and 24 °C. In order to determine the influence of a steel rebar, the sensor was located at 0, 14 and 28 mm (using pieces of 14 mm thick plywood) from a 25.4 mm diameter steel rebar. The thickness of the plywood used to separate the sensor from the steel rebar was 14 mm. In both cases, the magnetic field changes were measured with a gaussmeter and the corresponding resonant frequency determined.

## 3. Results and Discussion

### 3.1. Fresh Cement Pastes

**Figure 7.**
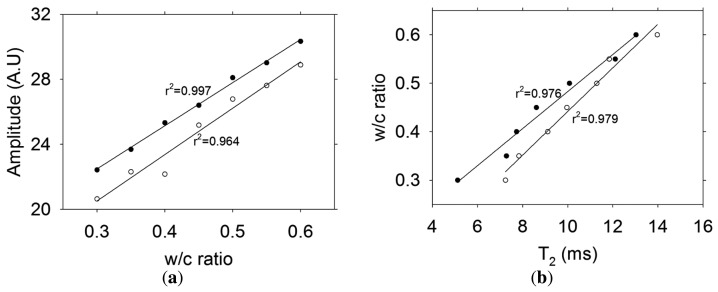
(**a**) Relationship between w/c ratio and NMR signal amplitude of cement pastes; (**b**) Relationship between T_2_ relaxation times and w/c ratio of cement pastes. The symbols indicate: ● ordinary Portland cement, ○ white Portland cement.

Figure 7a presents the relationship between the NMR signal and the water-to-cement ratio (w/c) by weight of fresh grey and white cement pastes. As expected, there is a linear relationship between the signal amplitude and the amount of water present in the cement pastes. Figure 7b shows the relationship between T_2_ and the w/c ratio for the fresh cement pastes. It is observed that T_2_ increases with increasing w/c ratio. This is in agreement with the fact that the w/c ratio is related to the distance between cement particles [14]. That is, in high w/c ratio pastes the cement particles are more separated than in low w/c ratio pastes. For the same w/c ratio, an enhanced relaxation produced by the higher amount of paramagnetic impurities (mainly iron) found in ordinary Portland cement (Table 1), is reflected in shorter T_2_ values with respect to white cement. From these results, it is seen that both approaches (signal amplitude and T_2_ values) would make it possible to determine the actual w/c ratio in fresh cement paste or concrete.

### 3.2. Hardened Cement Pastes

**Figure 8.**
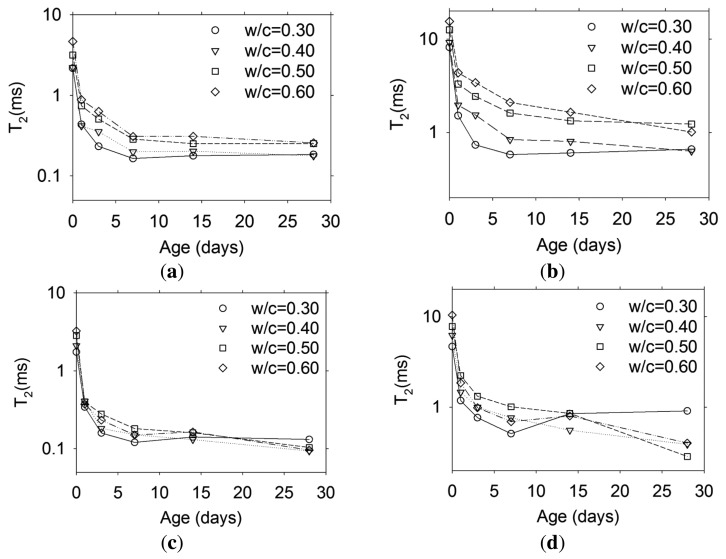
Evolution of the short and long lifetime components for (**a**) white cement-short T_2_; (**b**) white cement-long T_2_; (**c**) grey cement-short T_2_; and (**d**) grey cement-long T_2_.

Increasing hydration times cause the microstructure to change as long as there is enough water to support the hydration process [1]. Figure 8 shows the T_2_ variation with hydration time for the two types of cement pastes at different w/c ratios. It is observed that the short and long lifetime components, corresponding to gel and capillary pores [15], respectively, decrease with increasing age due to pore refinement caused by cement hydration. In Figure 8d it is interesting to note an increase in the long lifetime component only for the cement paste with w/c ratio of 0.30. Since this sample was sealed, there was no external water source that could help mitigate the autogenous shrinkage occurring in low w/c ratio materials [16] and micro-cracking was developed, thus increasing the surface-to-volume ratio of the porosity.

### 3.3. Performance of the Sensors in Hardened Hydraulic Concrete

**Figure 9.**
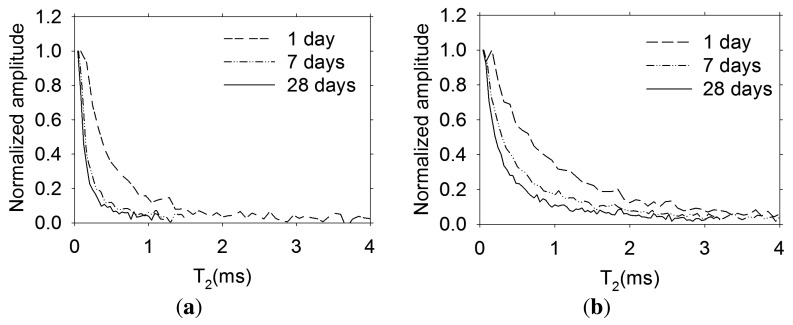
Normalized transverse magnetization decay measured with the embedded sensors in concrete (**a**) w/c ratio = 0.30 and (**b**) w/c = 0.60.

The normalized transverse magnetization decay at 1, 7 and 28 days of age obtained with the sensors embedded in concrete are shown in Figure 9. A more rapid decay in the specimen prepared with concrete with a w/c ratio = 0.30 is observed. This is expected, since the pores are finer with respect to the higher w/c ratio concrete.

Comparing the SNR of the signal obtained when using the new design in concrete with w/c ratio of 0.60 (SNR = 101 for 8192 scans) to the one reported in reference [10], which was mortar with w/c ratio of 0.60 (SNR= 27.3 for 8192 scans), there is an improvement factor of about 4. This increase in sensitivity was a result of a higher magnetic field (0.38 Tesla), homogeneity and larger sensitive volume due to the nature of the magnet arrangement with respect to the unilateral design (0.24 Tesla).

**Figure 10.**
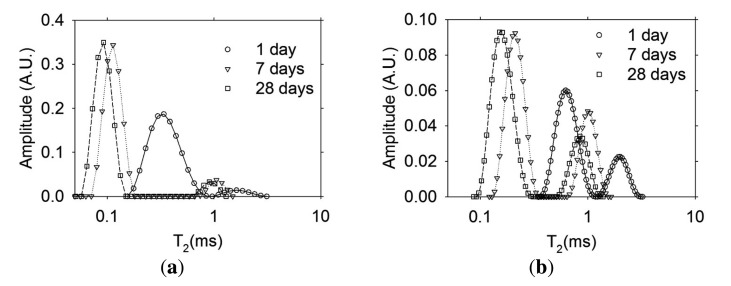
Inverse Laplace Transformation of the transverse magnetization decay measured with the embedded sensors in concrete (**a**) w/c ratio = 0.30; and (**b**) w/c = 0.60.

Figure 10 shows the Inverse Laplace Transformation of the transverse magnetization decay data [17] shown in Figure 9. As expected, the T_2_ distributions indicate that a finer porosity is obtained in the concrete with w/c ratio of 0.30 compared to the concrete with w/c ratio of 0.60 (lower T_2_ values in concrete with w/c of 0.30). On the other hand, in concrete with w/c ratio of 0.30, it is only possible to resolve one peak due to the quality of the NMR signal obtained. However, in the concrete with w/c ratio of 0.60, the signal obtained was of better quality and the ILT resolves the two types of pores known in cement-based materials: capillary and gel pores [1].

The size of the gel pores in a hydrating cement paste are in the order 1 to 3 nm, whereas the size of the capillary pores depends on the w/c ratio and the degree of hydration. The size of capillary pores are in the order of 10 to 200 nm for a hydrated cement paste having a w/c ratio of 0.60, and from 10 to 90 nm for a paste with a w/c ratio of 0.30 [1]. The materials used in the present study would have gel and capillary pores sizes similar to those mentioned before.

The NMR relaxation times are sensitive to the pore structure of materials and decrease as the pore size also decreases. These times vary widely for chemically bound hydrogen, hydrogen adsorbed on the surfaces, and hydrogen in water confined in small pores. It is known that the transverse relaxation rate 1/T_2_ is proportional to the surface to volume ratio (S/V) of the pore system, as expressed by Equation (1) [18]:

(1) $$\frac{1}{T_{2}}=\rho \frac{S}{V}$$

where ρ = T_2_ surface relaxivity (T_2_ relaxing strength of the pore surfaces), varies with the chemical composition of the pore surface.

**Figure 11.**
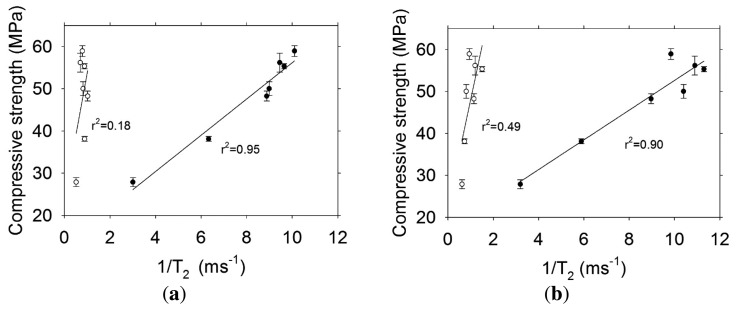
Transverse relaxation rate versus compressive strength for concrete w/c = 0.30 for (**a**) Sensor 1 and (**b**) Sensor 2. The symbols indicate: ● Short lifetime component, ○ Long lifetime component.

It is a common practice in magnetic resonance to use T_2_ as a proxy for pore size and not to convert the data to actual pore size. Figure 11 shows the transverse relaxation rate (1/T_2_) versus compressive strength of concrete cylinders with w/c = 0.30. The transverse magnetization decay was better fit to a bi-exponential decay, therefore short and long lifetime components were obtained. For both sensors it is observed that the highest correlation is obtained with the short lifetime component (r^2^ ≥ 0.95). The poor correlation observed with the long lifetime component, which is related to large capillaries, might be affected by internal micro-cracking caused by significant autogenous shrinkage occurring in materials with low w/c ratio [16,18].

**Figure 12.**
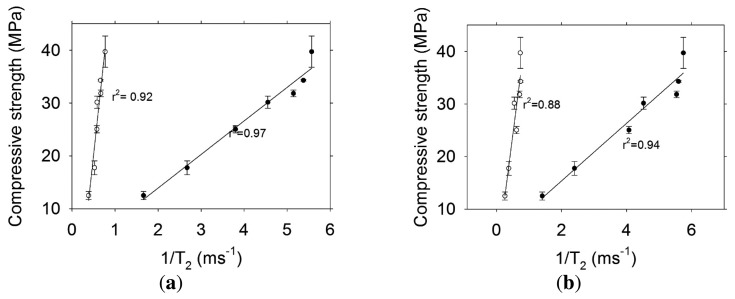
Transverse relaxation rate versus compressive strength for concrete w/c = 0.60 for (**a**) Sensor 3 and (**b**) Sensor 4. The symbols indicate: ● Short lifetime component, ○ Long lifetime component.

Figure 12 shows the results obtained for the relationship between the transverse relaxation rate and the compressive strength of concrete with w/c ratio = 0.60. In this case, both components (short and long lifetime) are highly correlated to mechanical strength. The highest r^2^ is obtained with the short lifetime component (r^2^ > 0.97). The effects of autogenous shrinkage in concrete with high w/c ratio are negligible [19], therefore micro-cracking should not have any significant effect on these specimens and the long lifetime component observed has information only from capillary porosity.

**Figure 13.**
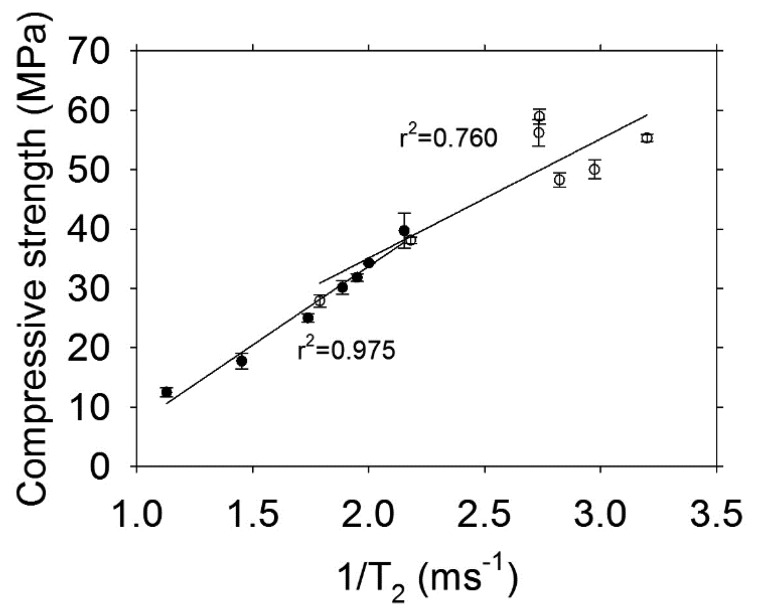
Transverse relaxation rate obtained with the Oxford Instruments Maran DRX-HF 12/50 system versus compressive strength. The symbols indicate: ● Concrete w/c = 0.60, ○ Concrete w/c = 0.30.

The correlations obtained from the T_2_ measurements undertaken with the Oxford Instruments Maran DRX-HF 12/50 are shown in Figure 13. The r^2^ are similar to those obtained with the sensors. Because the samples used small pieces taken from destructive testing, extensive cracking was evident by visual examination. This condition affected the long T_2_ and the correlation in concrete with low and high w/c ratio had a very low r^2^.

### 3.4. Influence of Temperature and Reinforcing Steel Bars on Sensor Resonant Frequency

Table 5 shows the frequency variation due to changes in ambient temperature or by the presence of a steel rebar.

It is observed that the frequency decreases with increasing temperature while the frequency increases with reduced distance of the sensor to the steel rebar. These changes in frequency can be easily accommodated with the external tuning circuit. Based on the limited data obtained related to the influence of temperature on the sensor's magnetic field, a reasonable range of variation could be from 15 to 30 °C, which would produce changes in frequency that can be accommodated with the external tuning circuit that has a range of 1 MHz. Since the effects on frequency caused by the presence of steel bars were measured at 14 and 28 mm (one and two times the thickness of the plywood used to separate the steel from the sensor), 28 mm might be the minimum distance because the presence of the steel does not affect the magnetic field of the sensor. At shorter distances and depending on the steel bar diameter, it might create significant magnetic field gradients due to the corrugated surface of the reinforcing steel used in the construction industry. With respect to the direction, because the sensor is considered as a point sensor, it would seem to have no natural direction.

## 4. Conclusions

An improved miniature embeddable NMR sensor for use in cement-based materials was designed, built and characterized. Changes in frequency due to temperature variations and the presence of steel rebars can be accommodated using an external tuning circuit. The sensor was successfully used to detect water in fresh cement pastes and to monitor porosity refinement in hardened cement pastes and concretes containing ordinary materials routinely used in the construction industry. A linear relationship exists between the relaxation rate and the compressive strength of concrete mixtures at low and high w/c ratios. The results demonstrate that practical applications are possible and they will be pursued.

## Figures and Tables

**Table 1.** CPMG parameters used to obtain the SNR for each sensor using a polymer phantom, which has a long lifetime component of 20 ms and a short lifetime component of 4.5 ms.

**Sensor**	**Frequency (MHz)**	**No. of Scans**	**Echo Time (μs)**	**No. of Echoes**	**Repetition Time (ms)**	**pw_90°_(μs)**	**RF Power (Watts)**	**Acquisition Time (min)**	**SNR**
S1	15.47	4,096	200	300	500	7	100	34	139
S2	15.39	4,096	200	300	500	6	100	34	173
S3	15.48	4,096	200	300	500	6	100	34	148
S4	15.17	4,096	200	300	500	6	100	34	160

**Table 2.** CPMG parameters used for the measurements in cement pastes and concretes.

**Material**	**No. of** **Scans**		**Echo** **Time/μs**		**No. of** **Echoes**		**Repetition** **Time/ms**		**pw_90°_/μs**	**RF** **power/W**	**Acquisition** **Time/min**	
				
	**Min**	**Max**	**Min**	**Max**	**Min**	**Max**	**Min**	**Max**			**Min**	**Max**
White cement paste	1,024	4,096	50	200	60	450	250	500	6	100	8.5	17.0
Grey cement paste	1,024	4,096	40	200	30	250	250	500	6	100	8.5	17.0
Concrete w/c = 0.30	4,096	8,192	36	80	30	50	250	500	6	100	34.0	34.0
Concrete w/c = 0.60	4,096	8,192	40	120	50	80	250	500	6	100	34.0	34.0

**Table 3.** Oxide composition of the cements and fine aggregate used to prepare the cement pastes and concrete mixtures. LOI means mass loss on ignition.

**Oxide (%)**	**Al_2_O_3_**	**CaO**	**Fe_2_O_3_**	**K_2_O**	**MgO**	**MnO**	**Na_2_O**	**P_2_O_5_**	**SiO_2_**	**TiO_2_**	**LOI**
White Portland cement	3.87	67.23	0.26	0.34	0.60	N.D.	0.08	N.D.	21.95	0.07	5.76
Ordinary Portland cement	3.69	58.70	3.97	0.31	1.58	0.10	0.18	0.09	18.75	0.17	5.44
Fine Aggregate	11.91	1.80	1.26	2.66	0.60	0.06	3.83	0.12	72.91	0.07	0.97

**Table 4.** Mixture proportions used to prepare 1 m^3^ of concrete.

**Materials**	**Water-to-Cement ratio, by Weight**	

	**w/c = 0.30**	**w/c = 0.60**
Coarse aggregate (Kg)	948	837
Fine aggregate (Kg)	671	794
Ordinary Portland cement (Kg)	519	345
Water (Kg)	184	232
Superplasticizer (L)	4.67	-

**Table 5.** Effect of ambient temperature and distance to a steel rebar on the frequency of the sensor.

**Temperature (°C)**	**Frequency (MHz)**
17.5	15.967
20.0	15.900
24.0	15.883
Distance (mm)	Frequency (MHz)
≥28.0	15.883
14.0	16.133
0.0	ND

## References

[1] Mehta P.K., Monteiro J.M. (2006). Concrete, Microstructure, Properties and Materials.

[2] ASTM F 2170-02 Standard Test Method for Determining Relative Humidity in Concrete Floor Slabs Using In-Situ Probes. http://www.astm.org/Standards/F2170.htm.

[3] Carino N.J. (1997). Concrete Construction Engineering Handbook.

[4] Blinc R., Dolinsek J., Lahajnar G., Sepe A., Zupancic I., Zumer S., Milia F., Pintar M.M. (1988). Spin-lattice relaxation of water in cement gels. J. Phys. Sci..

[5] Jehng J.Y., Sprague D.T., Halperin W.P. (1996). Pore structure of hydrating cement paste by magnetic resonance relaxation analysis and freezing. Magn. Reson. Imaging.

[6] Apih T., Lahajnar A., Sepe A., Blinc R., Milia F., Cvelbar R., Emri I., Gusev B.V., Titova L.A. (2001). Proton spin-lattice relaxation study of the hydration of self-stressed expansive cement. Cem. Concr. Res..

[7] Blumich B., Blumler P., Eidmann G., Guthausen A., Haken R., Schmitz U., Saito K., Zimmer G. (1998). The NMR-mouse: Construction, excitation, and applications—application to NMR imaging of elastomers. Magn. Reson. Imaging.

[8] Boguszynska J., Brown M.C.A., McDonald P.J., Mitchell J., Mulheron M., Tritt-Goc J., Verganelakis D.A. (2005). Magnetic resonance studies of cement based materials in inhomogeneous magnetic fields. Cem. Concr. Res..

[9] Manz B., Coy A., Dykstra R., Eccles C.D., Hunter M.W., Parkinson B.J., Callaghan P.T. (2006). A mobile one-sided NMR sensor with a homogeneous magnetic field: The NMR-MOLE. J. Magn. Reson..

[10] Cano-Barrita P.F.J., Marble A.E., Balcom B.J., García J.C., Masthikin I.V., Thomas M.D.A., Bremner T.W. (2009). Embedded NMR sensors to monitor evaporable water loss caused by hydration and drying in Portland cement mortar. Cem. Concr. Res..

[11] Díaz-Díaz F. (2013). Sensor Miniatura de Resonancia Magnética Nuclear (NMR) de Bajo Costo Para Caracterizar de Manera no Destructiva Materiales Basados en Cemento (in Spanish). Master's Thesis.

[12] Meiboom S., Gill D. (1958). Modified spin–echo method for measuring nuclear relaxation times. Rev. Sci. Instrum..

[13] Ludwik R., Bretchko P. (2000). RF Circuit Design Theory and Applications.

[14] Bentz D., Aitcin P.C. (2008). The hidden meaning of water-cement ratio. Concr. Int..

[15] Halperin W.P., Jehng J.Y., Song Y.Q. (1994). Application of spin-spin relaxation to measurement of surface area and pore size distributions in a hydrating cement paste. Magn. Reson. Imaging.

[16] Tazawa E., Miyazawa S. (1995). Experimental study on mechanism autogenous shrinkage of concrete. Cem. Concr. Res..

[17] Borgia G.C., Brown R.J.S., Fantazzini P. (1998). Uniform-penalty inversion of multiexponential decay data. J. Magn. Reson..

[18] Coates G., Xiao L., Prammer M. (1999). NMR Logging Principles and Applications.

[19] Aitcin P.C. (1999). Demystifying autogenous shrinkage. Concr. Int..

